# Detection and imaging of Hg(II) in vivo using glutathione-functionalized gold nanoparticles

**DOI:** 10.3762/bjnano.13.46

**Published:** 2022-06-23

**Authors:** Gufeng Li, Shaoqing Li, Rui Wang, Min Yang, Lizhu Zhang, Yanli Zhang, Wenrong Yang, Hongbin Wang

**Affiliations:** 1 Key Laboratory of Resource Clean Conversion in Ethnic Regions, School of Chemistry and Environment, Yunnan Minzu University, Kunming 650500, P. R. China; 2 School of Life and Environmental Sciences, Deakin University, Waurn Ponds, Victoria 3216, Australia

**Keywords:** cell imaging, fluorescence probes, glutathione, gold nanoparticles, mercury ions, rhodamine 6G derivatives

## Abstract

The optical and biological properties of functionalized gold nanoparticles (GNPs) have been widely used in sensing applications. GNPs have a strong binding ability to thiol groups. Furthermore, thiols are used to bind functional molecules, which can then be used, for example, to detect metal ions in solution. Herein, we describe 13 nm GNPs functionalized by glutathione (GSH) and conjugated with a rhodamine 6G derivative (Rh6G2), which can be used to detect Hg(II) in cells. The detection of Hg^2+^ ions is based on an ion-catalyzed hydrolysis of the spirolactam ring of Rh6G2, leading to a significant change in the fluorescence of GNPs-GSH-Rh6G2 from an “OFF” to an “ON” state. This strategy is an effective tool to detect Hg^2+^ ions. In cytotoxicity experiments, GNPs-GSH-Rh6G2 could penetrate living cells and detect mercury ions through the fluorescent “ON” form.

## Introduction

Metal nanoparticles have been widely used in the development and construction of sensor systems and drug carriers due to their excellent biocompatibility, large specific surface area, and remarkable photoelectric properties [1–3]. Among them, gold nanoparticles (GNPs) have been frequently employed for drug delivery, sensing, imaging, and photodynamic therapy owing to their high extinction coefficient, distinct optical properties, excellent biocompatibility, and low toxicity [4–9]. Another advantage of GNPs is that different shapes and sizes can be obtained during synthesis through changing reducing agents and reaction conditions [10]. The surface chemistry of GNPs is modified via ligands with functional groups such as thiol (–SH), amino (–NH_2_), and carboxyl (–COOH) groups [11–14]. The surface of GNPs can be easily modified with good stability. Thus, they can penetrate the cell membrane and selectively interact with target biomolecules in cells [15–18].

So far, a variety of functionalized GNPs, whose properties were tuned by specific molecules, has been reported. For example, Coelho et al. reported that pegylated gold nanoparticles were combined with doxorubicin and varlitinib [19]. The modified pegylated gold nanoparticles could not only reduce the toxicity to normal cells but also improve the inhibitory effect on cancer cells. In another work, Basu et al. designed a novel sensing system using DNA-functionalized GNPs. GNPs have a strong binding affinity to phosphate and sugar groups in DNA [20]. The combined GNPs-DNA has unique physicochemical properties and was used to detect Mg^2+^. Furthermore, Liu et al. synthesized a novel probe using gold nanoparticles modified by rhodamine B isothiocyanate and poly(ethylene glycol) (RBITC-PEG-GNPs) [21]. A cytotoxicity assay showed that a cell viability of 95–100% was maintained during the incubation with RBITC-PEG-GNPs with different concentrations from 0 to 80 nM. Thus, surface modifications of GNPs are highly attractive for both environmental monitoring and biological applications.

Surface modification of GNPs by using self-assembled monolayers (SAMs) [22] is one of the most attractive strategies to enhance their sensing performance. The surface of GNPs can be modified through the interaction between covalent and non-covalent bonds due to the smaller steric hindrance [23–25]. The surface of GNPs can be modified by Au–S bonds with molecules containing thiol groups, such as cysteine [26–28], 3-mercaptopropionic acid [29], and homocysteine [30]. Also, they easily conjugate with drug molecules and fluorescent dyes [24]. Recently, we developed a novel Cu(II)-triggered release system with gold nanoparticles surface-modified with ʟ-cysteine for molecular delivery and imaging in cells [31]. Well dispersed GNP–ʟ-cysteine was conjugated with Rh6G2 (GNP–ʟ-Cys–Rh6G2) for a molecular release system. After adding Cu(II), we observed a switching of the GNP–ʟ-Cys–Rh6G2 fluorescence from “OFF” to “ON” with high stability.

Furthermore, it is worth noting that glutathione (GSH) contains a thiol and an amino group. It can not only conjugate to the nanoparticle surfaces through the thiol group, but also combine with related molecules via its other groups [32]. GSH-modified GNPs can improve the biocompatibility of GNPs [33–35]. Therefore, GSH can be used to modify the surface of GNPs for improving their stability, safety, and biocompatibility. However, in order to enhance the capabilities of the GSH-modified GNPs, additional modification strategies are needed.

In this study, rhodamine 6G derivative conjugated to GSH-modified GNPs (GNPs-GSH-Rh6G2) was designed and synthesized in order to effectively tune the properties of GSH-functionalized GNPs for the detection of Hg^2+^ and cell imaging. We chose rhodamine 6G because of its excellent light stability, high fluorescence quantum yield, and good biocompatibility [36–37]. The functionalized GNPs have excellent selectivity for Hg^2+^. Furthermore, to evaluate the imaging effects of functionalized GNPs in cells, GNPs-GSH-Rh6G2 was incubated in HeLa cells. We expect that this new system based on the molecular regulation of functionalized GNPs can have potential applications in pollution monitoring, biosensing, and cellular imaging.

## Results and Discussion

### Synthesis and spectral signature of GNPs-GSH-Rh6G2

**Figure 1:**
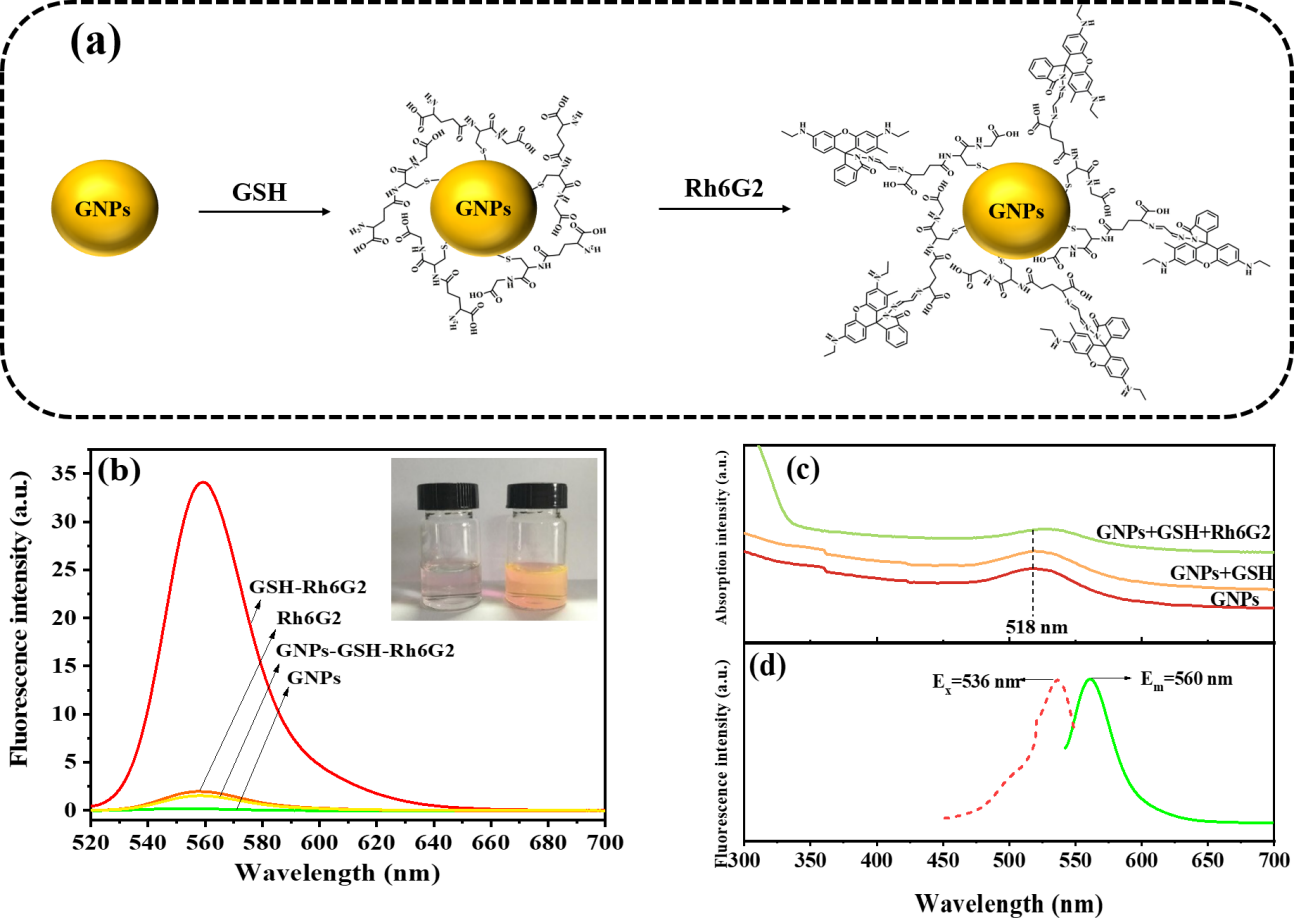
(a) Synthesis of functionalized GNPs-GSH-Rh6G2; (b) fluorescence spectra of GNPs, Rh6G2, GSH-Rh6G2, and GNPs-GSH-Rh6G2 (inset: the left bottle and the right bottle contain GNPs-GSH-Rh6G2 and GSH-Rh6G2, respectively); (c) UV–vis spectra of GNPs, GNPs-GSH, and GNPs-GSH-Rh6G2; (d) fluorescence excitation and emission spectra of GNPs-GSH-Rh6G2.

As shown in Figure 1a, 13 nm GNPs were synthesized by a previously reported protocol [38], then the surface of GNPs was modified with GSH to form SAM-modified GNPs. Subsequently, the carbonyl group of Rh6G2 was conjugated with the amino groups of GSH-modified GNPs via Schiff base reaction in methanol solution [39–40].

It is worth mentioning here that the pH value of the reaction systems played an important role in the synthesis of GSH-Rh6G2. Rh6G2, as an ideal candidate for controlled-release molecular systems, shows little fluorescence (Figure 1b). In the absence of GNPs, the conjugation of GSH-Rh6G2 yields obvious fluorescence. The thiol group in GSH and the aldehyde group in Rh6G2 undergo an addition reaction, followed by the formation of unstable intermediates [41]. Ultimately, the carboxyl groups of GSH will provide an acidic microenvironment so that a hydrolysis reaction will occur resulting in the formation of RGCOOH (Figure S1, Supporting Information File 1) [42]. The final product of the reaction between Rh6G2 and GSH was proved by TOF-MS analysis (Figure S2, Supporting Information File 1). By adjusting the pH value from an unadjusted slightly acidic environment to pH 7, a significant decrease in the fluorescence of GSH-Rh6G2 was observed (Figure S3, Supporting Information File 1).

GSH binds to GNPs via the thiol moiety, leading to an exposure of the amino group. Functional molecules can be directly bound via GSH, providing a stable environment based on the GNP nanostructure.

The UV–vis absorption and fluorescence spectra of GNPs-GSH-Rh6G2 are shown in Figure 1b,c. Figure 1c shows a strong absorption peak of 13 nm GNPs with the typical plasmon band of gold nanoparticles at 518 nm. However, the absorption peaks of GNPs-GSH and GNPs-GSH-Rh6G2 were slightly redshifted from 518 to 522 and 536 nm, respectively, caused by a change in the local dielectric environment and the plasmonic absorption bands of GSH and GSH-Rh6G2-modified GNPs [43–44]. The maximum fluorescence absorption peak of GNPs-GSH-Rh6G2 is at 536 nm, whereas the emission peak is at 560 nm (Figure 1d). The excitation of GNPs-GSH-Rh6G2 was examined in order to further evaluate its unique emission features. Figure S4 (Supporting Information File 1) shows the emission spectra of GNPs-GSH-Rh6G2 at various excitation wavelengths ranging from 486 to 536 nm. The fluorescence emission wavelength of GNPs-GSH-Rh6G2 appears to exhibit a non-excitation property when the excitation wavelength is changed. Furthermore, the fluorescence intensity of GNPs-GSH-Rh6G2 increased with increasing excitation wavelength, but no complete peak appeared at excitation wavelengths of 526 and 536 nm. Therefore, the emission intensity of the GNPs-GSH-Rh6G2 at 560 nm under excitation at 516 nm was chosen as the signal of the GNPs-GSH-Rh6G2.

### Characterization of GNPs-GSH-Rh6G2

**Figure 2:**
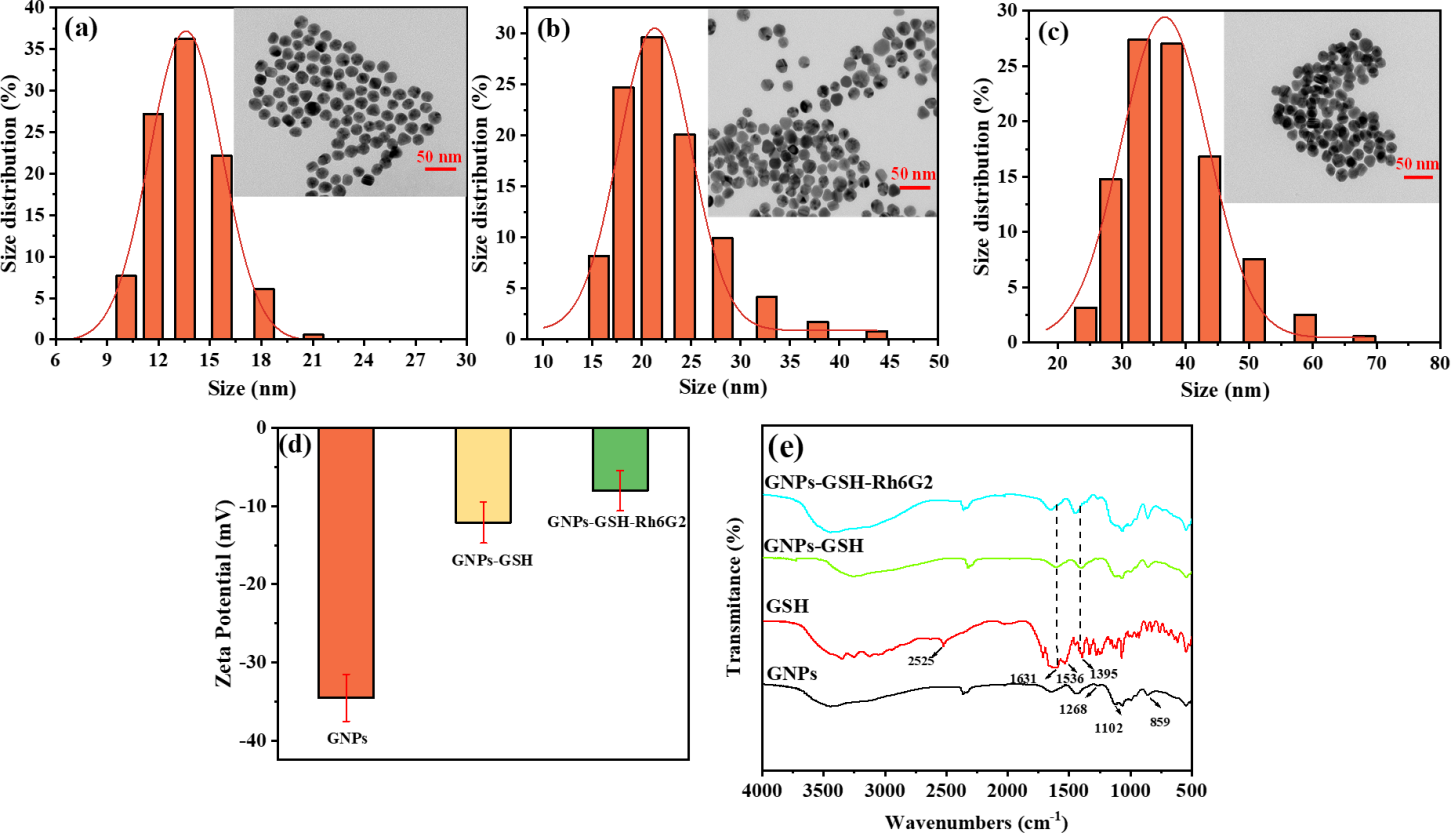
DLS of (a) GNPs, (b) GNPs-GSH, and (c) GNPs-GSH-Rh6G2 (insets: TEM images); (d) zeta potential of GNPs, GNPs-GSH, and GNPs-GSH-Rh6G2; (e) FTIR spectra of GNPs, GSH, GNPs-GSH, and GNPs-GSH-Rh6G2.

To confirm the formation of GNPs-GSH-Rh6G2, transmission electron microscopy (TEM) was carried out (insets of Figure 2a–c). The TEM image in Figure 2a shows that pure GNPs were well dispersed with a diameter of about 13 nm according to DLS measurements. GSH-modified GNPs displayed similar morphology and sizes (Figure 2b) [6,45]. When GNPs-GSH were further modified with Rh6G2, the size of the modified GNPs was slightly increased, and no aggregation occurred (Figure 2c) [46]. Furthermore, the surface charges of GNPs, GNPs-GSH, and GNPs-GSH-Rh6G2 are shown in Figure 2d. Once GNPs were modified with GSH, the surface potential increased from −34.5 to −12.1 mV due to the positive surface charges of GSH. After further modifying with Rh6G2, the zeta potential increased to −8 mV. These results indicated that the GSH and Rh6G2 were successfully bound to the surface of GNPs.

FTIR spectra of GNPs, GSH, GNPs-GSH, and GNPs-GSH-Rh6G2 are presented in Figure 2e. As citrate ions are attached on the surface of GNP, C=O, and C–O stretching vibration modes occur at 1655 and 1443 cm^−1^, respectively. The peaks of GSH at 1650 and 1400 cm^−1^ were found in the IR spectrum of GNPs-GSH, which was attributed to the stretching vibration and the asymmetric stretching vibration of –COO^−^. The stretching vibration of S–H disappeared in GNPs-GSH due to the formation of Au–S bonds [47]. These results proved that GSH was immobilized on the surface of GNPs. Rh6G2 peaks at 1640 and 1072 cm^−1^ were found on GNPs-GSH-Rh6G2, which were ascribed to C=N and C–N stretching vibrations. The peak intensity was also more obviously enhanced than that of GNPs-GSH, indicating that GNPs-GSH-Rh6G2 has been successfully prepared.

### Synthesis of GNPs-GSH-Rh6G2

**Figure 3:**
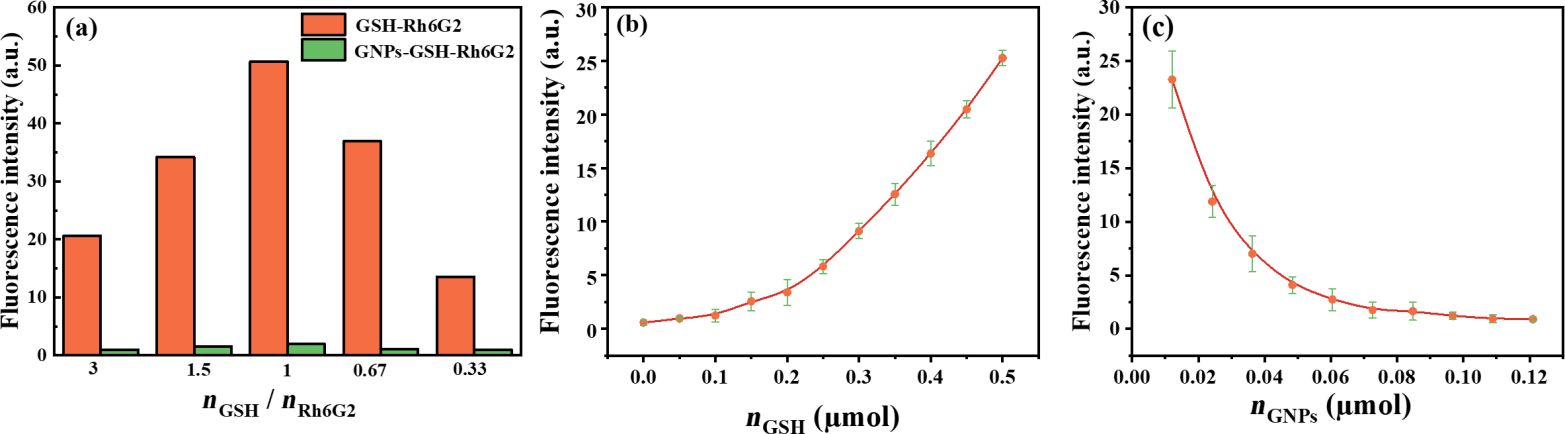
(a) Fluorescence intensity of GNPs-GSH-RH6G2 as function of the ratio between *n*_GSH_ and *n*_Rh6G2_ (*n*_GNPs_ is 0.0726 µmol); (b) fluorescence intensity of GNPs-GSH-Rh6G2 synthesized using different amounts of GSH (*n*_GNPs_ and *n*_RH6G2_ are 0.0726 µmol and 0.15 µmol, respectively); (c) fluorescence intensity of GNPs-GSH-Rh6G2 synthesized using different amounts of GNPs (*n*_GSH_ and *n*_RH6G2_ are 0.15 µmol).

In order to fabricate a robust and highly sensitive fluorescent probe, we optimized the synthesis conditions of GNPs-GSH-Rh6G2 including the molar ratio between GSH and Rh6G2 and the concentrations of GNPs and GSH. It was reported that the amino group in the GSH molecule and the aldehyde group in Rh6G2 undergo a cyclization reaction to form a thiazolidine structure [41]. Figure 3a shows that the fluorescence of GNPs-GSH-Rh6G2 reached the maximum when the molar ratio between GSH and Rh6G2 was 1, and the obtained molar ratio is consistent with the theoretical value of 1.

To investigate the effect of the concentration of GSH, different amounts of GSH were added. As shown in Figure 3b, when the amount of GSH is less than 0.15 µmol, there is basically no fluorescence, which is attributed to all GSH and Rh6G2 being conjugated on the GNPs surface such that there are no free GSH and Rh6G2 molecules capable of reacting with each other. GSH and GNPs were completely conjugated when the amount of GSH was 0.15 µmol. However, we observed that excess GSH reacts with Rh6G2 to form a thiazolidine that is easily hydrolyzed to generate fluorescence. Therefore, 0.15 µmol GSH was chosen for the surface modification of GNPs. Figure 3c shows that the fluorescence intensity gradually decreases and tends to be stable with increasing amounts of GNPs. When an amount of GNPs greater than 0.0726 µmol was added, GNPs-GSH-Rh6G2 exhibited little fluorescence, indicating that GNPs and GSH were fully conjugated. Furthermore, when GSH and Rh6G2 are combined, there is a strong characteristic absorption peak at 560 nm (Figure S5, Supporting Information File 1). However, when GNPs were added to GSH-Rh6G2, the fluorescence disappeared.

**Figure 4:**
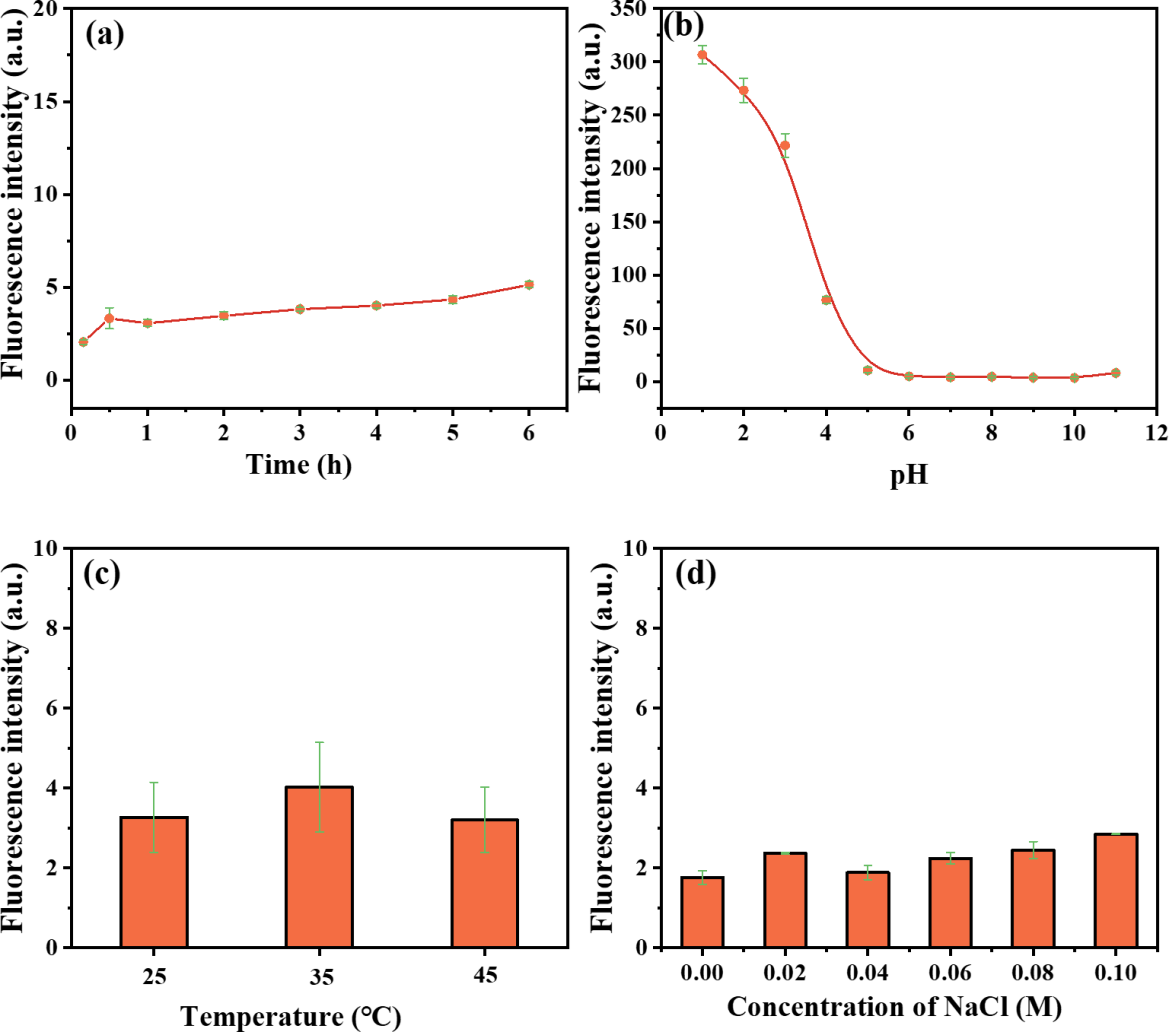
Fluorescence intensity of GNP-GSH-Rh6G2 in HEPES/CH_3_OH buffer (1:1 (v/v), 50 mM) as a function of (a) time, (b) pH value, (c) temperature, and (d) NaCl concentration.

Expectedly, we also found the fluorescence of GNPs-GSH-RH6G2 to be very weak in the first six hours as shown in Figure 4a. Furthermore, the pH value is critical regarding the fluorescence intensity. GNPs-GSH-Rh6G2 displayed a large change in the fluorescence intensity in the pH range from 1 to 5 (Figure 4b). This indicates that Rh6G2 tends to be protonated at low pH values, which enables ring reactions [48]. However, the fluorescence intensity of the GNPs-GSH-Rh6G2 remained stable in the range of pH 6–11 with a lower fluorescence baseline, which can help further studies in cells and organisms at pH 7. The effect of the temperature is shown in Figure 4c. There is little fluorescence in the temperature range of 25–45 °C. In addition, we also investigated the effect of the electrolyte solution (taking NaCl solution as an example) on the stability of GNPs-GSH-Rh6G2. As shown in Figure 4d, the fluorescence intensity of GNPs-GSH-Rh6G2 remained relatively stable when the concentration of electrolyte solution was increased. The GNPs-GSH-Rh6G2 have a lower fluorescence baseline in 0.10 M NaCl solution, which is important for the application in living organisms.

### Detection of Hg(II)

**Figure 5:**
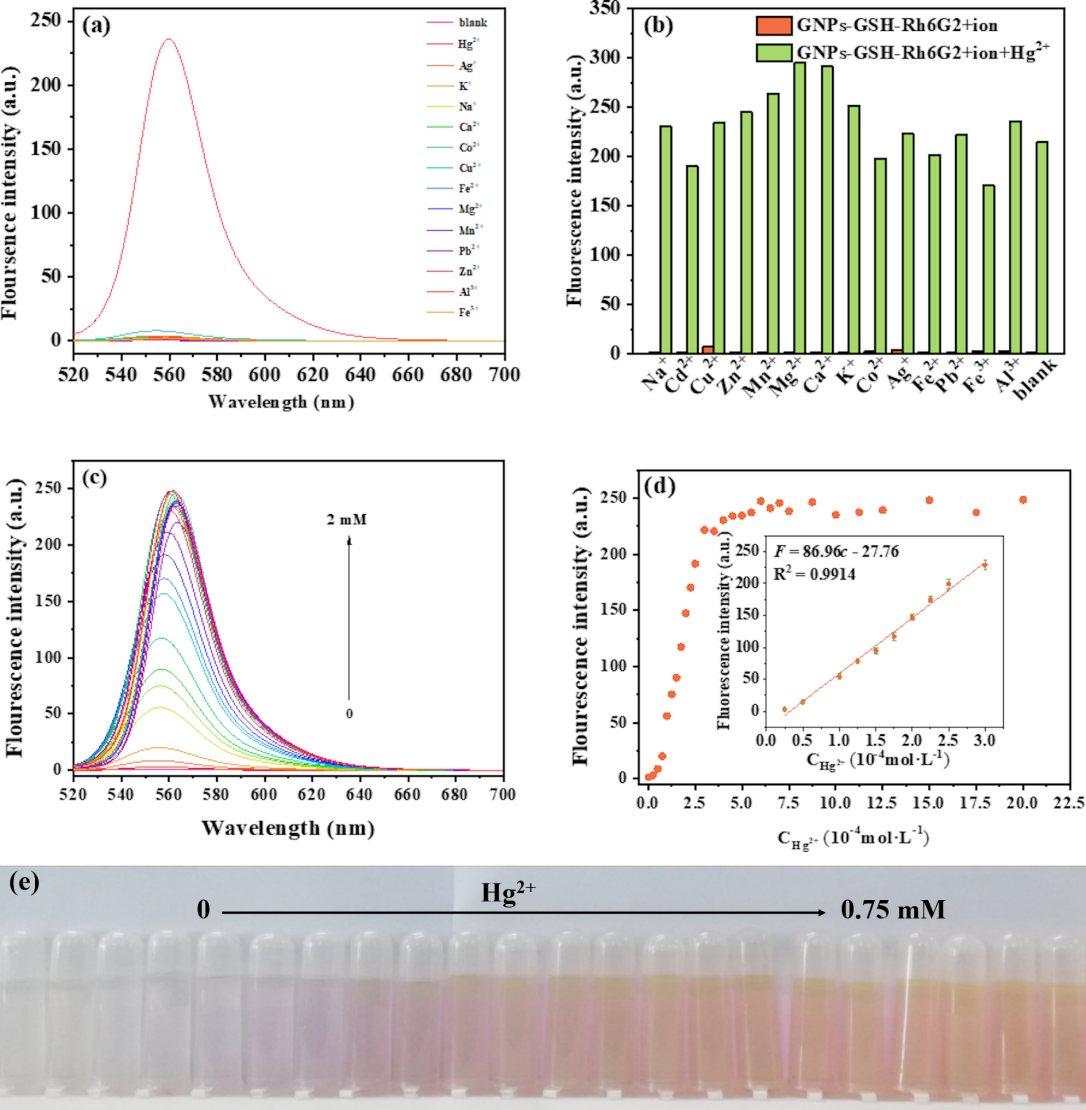
(a) Fluorescence spectra of GNPs-GSH-Rh6G2 with different ions (Hg^2+^, Na^+^, Cu^2+^, Zn^2+^, Mn^2+^, Mg^2+^, Ca^2+^, K^+^, Co^2+^, Ag^+^, Pb^2+^, Fe^3+^, and Al^3+^, concentration: 0.75 mM) in HEPES/CH_3_OH buffer (1:1 (v/v), 50 mM, pH 7); (b) fluorescence intensity at 560 nm of GNPs-GSH-Rh6G2 in the presence of Hg^2+^ (0.75 mM) and different metal ions (0.75 mM); (c) fluorescence spectra of GNPs-GSH-Rh6G2 in HEPES/CH_3_OH buffer (1:1 (v/v), 50 mM, pH 7) exposed to different concentrations of Hg^2+^; (d) fluorescence intensity at 560 nm as a function of the Hg^2+^ concentration; (e) photographs of GNPs-GSH-Rh6G2 containing different amounts of Hg^2+^.

We investigated the optical sensing properties of GNPs-GSH-Rh6G2 using fluorescence spectroscopy. To evaluate the specificity of GNPs-GSH-Rh6G2, a variety of cations, including Hg^2+^, Ag^+^, K^+^, Na^+^, Ca^2+^, Co^2+^, Cu^2+^, Fe^2+^, Mg^2+^, Mn^2+^, Pb^2+^, Zn^2+^, Al^3+^, and Fe^3+^, were examined. As shown in Figure 5a, except for Hg^2+^, there is little optical response. We also studied the fluorescence-triggered release of Hg^2+^ from GNPs-GSH-Rh6G2 in the presence of other cations (Figure 5b); none of the other ions has a substantial impact on the fluorescence-triggered release.

Figure 5c,d shows that the fluorescence intensity of GNP-GSH-Rh6G2 increased with an increase of Hg^2+^ concentration up to 0.75 mM. We observed a color change of GNPs-GSH-RH6G2 from colorless to pink when Hg^2+^ was added (Figure 5e). The fluorescence response showed a good linear relationship in the concentration range of 0.025–0.3 mM. The linear regression equation was *F* = 86.96*c* − 27.76 (*R*^2^ = 0.9914) (*c* represents Hg^2+^ concentration), and the limit of detection (LOD) was calculated to be 8.4 μM (*S*/*N* = 3). In Table 1 the analytical performance of the constructed sensors is compared with other reported methods and with sensing techniques based on GNPs. The results indicate that the proposed strategy has good selectivity and acceptable sensitivity compared to other non-GNP methods. It must be acknowledged that the detection window of GNPs-GSH-Rh6G2 is very wide (0.025–0.3 mM) but at the cost of a high LOD.

**Table 1:** Comparison of GNPs-GSH-Rh6G2 with other strategies for Hg^2+^ detection.

Materials	Linear range	LOD	Reference

AgNP-FA-PGE	10–25 μM	8.43 μM	[49]
AgN_P_O	0–30 μM	9.2 μM	[50]
SAC	10–50 μM	12.6 μM	[51]
RATU	0.1–90 μM	6.36 μM	[52]
GNPs	1–20 μM	1.44 μM	[53]
GNPs/ERGO	0.5–20 µg/L	0.06 µg/L	[54]
DNA-functionalized MoS_2_ nanosheet/GNP hybrid field-effect transistor	0–10 nM	0.1 nM	[55]
GNP-labeled ssDNA	0.1–0.5 nM	0.0015 nM	[56]
GNPs-GSH-Rh6G2	25–300 μM	8.4 μM	this work

### GNPs-GSH-Rh6G2 bioimaging in living cells

**Figure 6:**
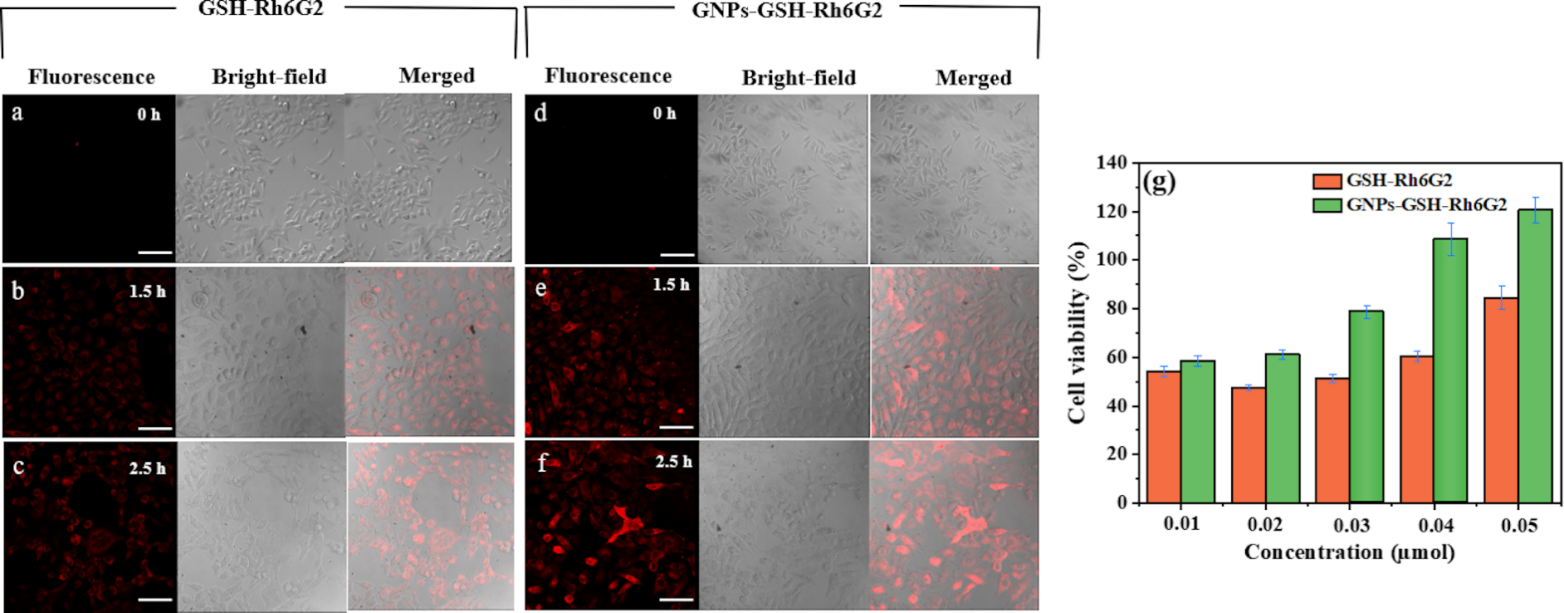
Real-time fluorescence imaging of HeLa cells treated with (a–c) GSH-Rh6G2 and (d–f) GNPs-GSH-Rh6G2 together with 100 µL with Hg^2+^ after different incubation times (0, 1.5 and 2.5 h). The scale bars are 100 µm. From left to right, the images represent fluorescence, bright-field, and merged-channel fluorescence imaging. (g) Evaluation of cytotoxicity on HeLa cells of GSH-Rh6G2 and GNPs-GSH-Rh6G2 at different concentrations (0.01, 0.02, 0.03, 0.04, and 0.05 µmol) after incubation for 24 h.

To study bioimaging of GSH-Rh6G2 and GNPs-GSH-Rh6G2 in living cells, confocal laser scanning microscopy was performed after different cell incubation times (0–2.5 h). As shown in Figure 6a and Figure 6d, when cultured HeLa cells were incubated with GSH-Rh6G2 and GNPs-GSH-Rh6G2 without Hg^2+^, there are no obvious intracellular fluorescence signals. After Hg^2+^ was added (10 µM) for 1.5 h, fluorescence in living cells was observed gradually (Figure 6b and Figure 6e), indicating that GSH-Rh6G2 and GNPs-GSH-Rh6G2 could enter the cells and that the release of RGCOOH was triggered by intracellular Hg^2+^. Importantly, we found that the cellular uptake of GNPs-GSH-Rh6G2 was higher than that of GSH-Rh6G2. This may be due to the introduction of gold nanostructures. Previous studies showed that GNPs-GSH-Rh6G2 permeate well into cells [48,57–58].

Cytotoxicity studies of nanomaterials are important to determine the effects of different components of the nanostructure [59–60]. The cytotoxicity of GSH-Rh6G2 and GNPs-GSH-Rh6G2 at different concentrations (0.01, 0.02, 0.03, 0.04, and 0.05 µmol) on HeLa cells was evaluated through CKK-8 assays. Figure 6g shows that the viability of HeLa cells increases with increasing concentration after 24 h of incubation with free GSH-Rh6G2 and GNPs-GSH-Rh6G2. In addition, GNPs-GSH-Rh6G2 demonstrates that gold nanoparticles can improve cell viability, indicating good biocompatibility [61].

**Figure 7:**
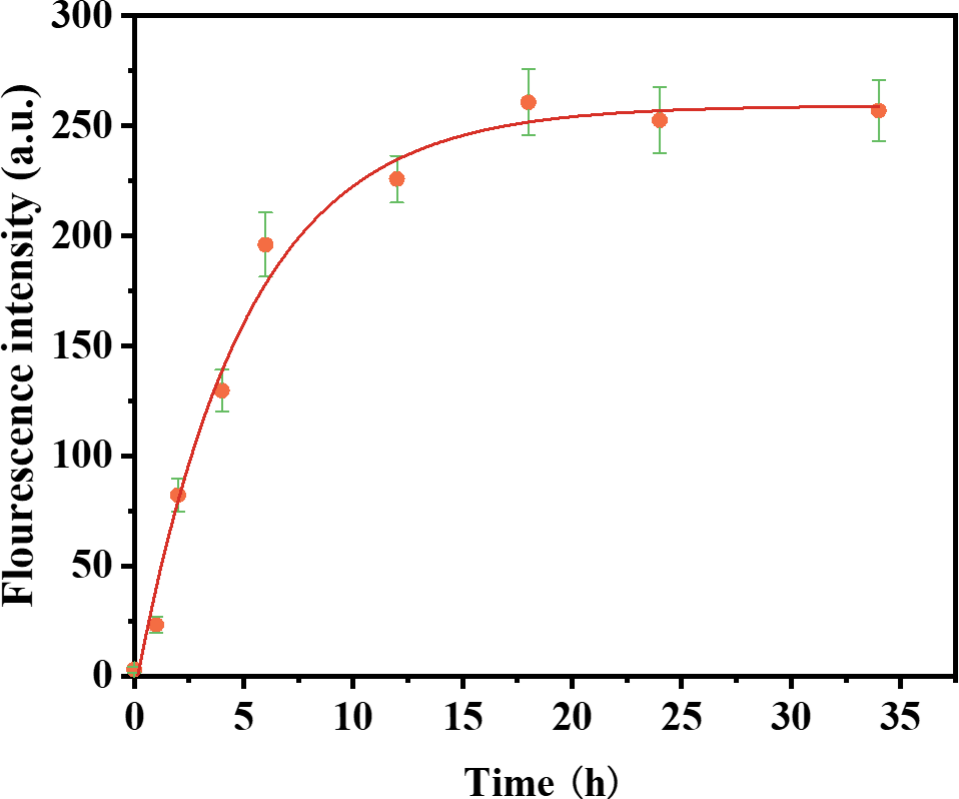
Fluorescence intensity of RGCOOH released from GNPs-GSH-Rh6G2 in the presence of Hg^2+^ within 35 h at room temperature in HEPES/CH_3_OH buffer (1:1 (v/v), 50 mM, pH 7).

To evaluate the release behavior of GNPs-GSH-Rh6G2, the triggered release of RGCOOH started when 30 μL Hg^2+^ was added to the solution. Figure 7 shows that the molecule was released within 20 h, which indicates a sustained release of RGCOOH from the Rh6G2-loaded GNPs-GSH.

**Figure 8:**
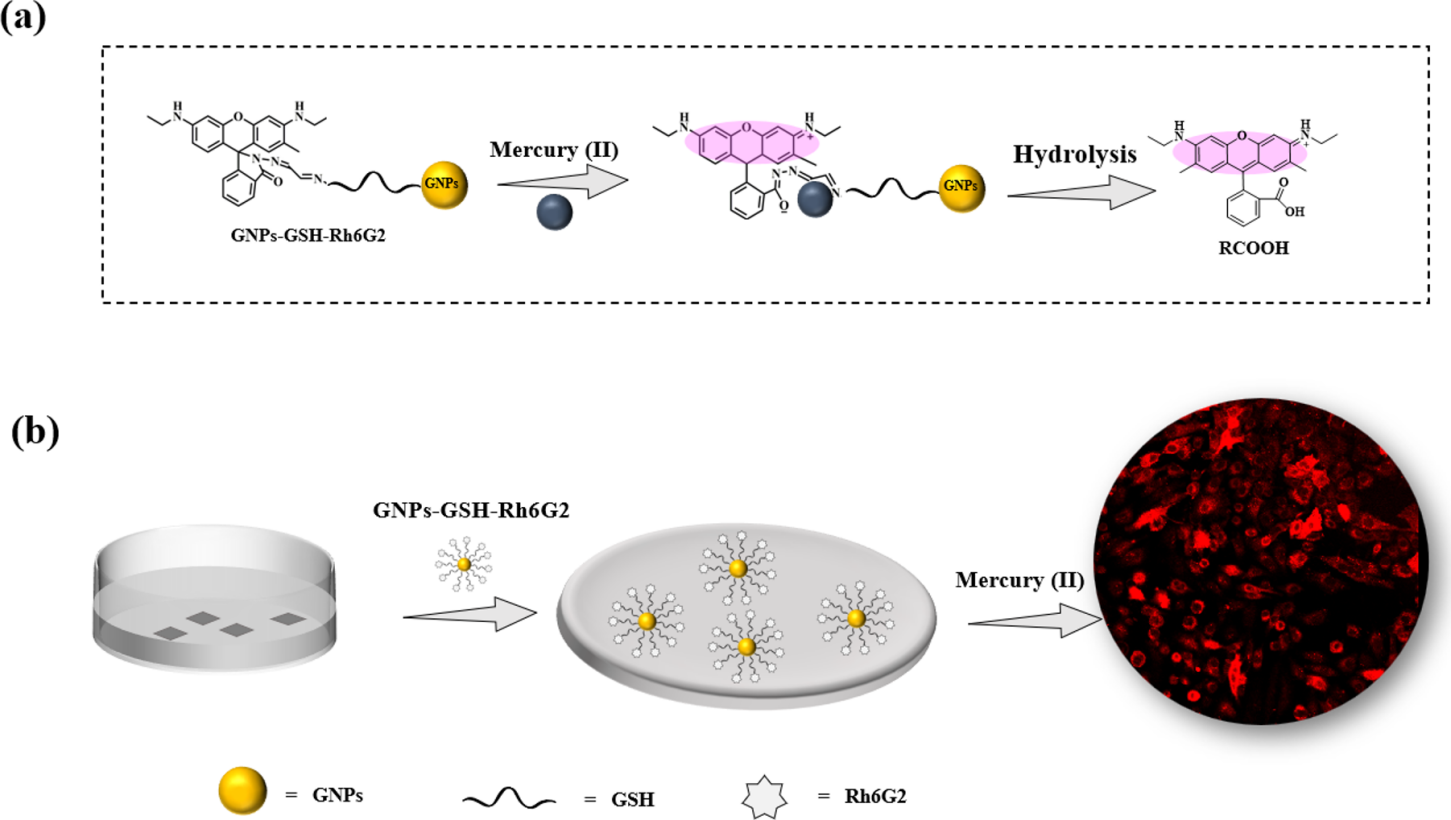
(a) Schematic illustration of the release mechanism of GNPs-GSH-Rh6G2 in the presence of Hg^2+^; (b) release of RGCOOH in cells through Hg^2+^.

Based on the experimental results, the release mechanism of GNPs-GSH-Rh6G2 by Hg^2+^ is illustrated in Figure 8a. Rh6G2, when conjugated with GNPs-GSH, does not exhibit fluorescence due to the closed spirolactam ring. Protons induce a weak fluorescence of the spirolactam framework at acidic pH through ring opening. Therefore, we suggest that in the presence of Hg^2+^ the formation of a Rh6G2–Hg^2+^ complex leads to ring opening, followed by the release of RGCOOH from the nanoparticle surface via hydrolysis, strongly increasing fluorescence emission [62].

GNPs-GSH-Rh6G2 did not generate detectable fluorescence signals after entering into the cells. However, when the cells were incubated with both GNPs-GSH-Rh6G2 and Hg^2+^, red fluorescence was obtained clearly (Figure 8b). This is because RGCOOH was released from GNPs when Hg^2+^ ions promoted a ring opening of the spirolactam moiety and hydrolysis occurred. Once the RGCOOH molecules were released and diffused into cells, they exhibited strong fluorescence (“ON”). So, by monitoring the fluorescence signal, it is possible to track molecules released into living cells.

## Conclusion

In this work, we conjugated GNPs with rhodamine 6G derivatives by surface functionalization of gold nanoparticles with glutathione. The conjugates can be used to generate a fluorescence signal in the presence of Hg^2+^. The fluorescence signal of GNPs-GSH-Rh6G2 in HEPES buffer solution shows a spectral response to the presence of metal ions, which illustrates the sensitivity and selectivity for Hg^2+^. Further, GSH-Rh6G2 and GNPs-GSH-Rh6G2 were employed in confocal microscopy experiments using Hela cells. The experiments showed that GNPs-GSH-Rh6G2 is more easily internalized into the cells and then releases RGCOOH. Notably, our strategy was able to significantly reduce cytotoxicity.

## Experimental

### Materials and instruments

Gold chloride hydrate (HAuCl_4_·4H_2_O) and rhodamine 6G (C_18_H_31_N_2_O_3_Cl) were provided from Sinopharm Chemical Reagent Co., Ltd. Trisodium citrate dihydrate and ʟ-glutathione in the reduced form were purchased from Shanghai Aladdin Bio-Chem Technology Co., Ltd. 2-[4-(2-Hydroxyethyl)piperazin-1-yl]ethane-1-sulfonic acid (HEPES) was purchased from Shanghai Mackin Biochemical Co., Ltd. Cervix carcinoma (HeLa cells) were purchased from Kunming Medical University. All reagents were of analytical grade.

TEM was carried out using a JEM-2100 transmission electron microscope (JEOL, Japan) at an accelerating voltage of 200 kV. UV–vis absorption spectra were obtained using a UV-2100 Spectrophotometer (Shimadzu, Japan). Fluorescence spectra were recorded using an F-7000 Fluorescence spectrophotometer (Hitachi, Japan). Mean particle size and the zeta potential were recorded using a Zetasizer Nano ZS90 (Malvern, UK). A Nicolet iS10 infrared spectrometer (Nicolet, USA) was used to gather FTIR spectra in a scanning range of 400–4000 cm^−1^. Fluorescence images of cells were acquired using an OLYMPUS CKX41 inverted fluorescence microscope (Olympus, Japan)/Leica SP5 laser scanning confocal microscope (Leica, Germany). Cell viability was measured by a PectraMax190 microplate reader (Molecular, USA). HPLC-MS was performed on an Agilent-ABQSTAR Pulsar (Agilent, Germany) with a high-resolution mass spectrometer.

### Synthesis of GSH-Rh6G and GNPs-GSH-Rh6G2

GNPs with a concentration of about 2.5 × 10^−4^ M were synthesized using a citrate reduction method [38]. 100 mL of HAuCl_4_ solution (0.24 mM) was boiled and stirred vigorously. To the mixture was swiftly added 3.5 mL of sodium citrate solution (0.34 mM) until the color changed from yellow to deep red. The mixture was brought to room temperature.

Compound Rh6G2 has been synthesized in a previous study [41] and its chemical structure was confirmed. The GSH- and Rh6G2-functionalized GNPs were prepared as follows: First, 150 μL of GSH stock solution (1 mM) prepared in deionized water was added into 300 μL of 13 nm GNPs solution for 0.5 h. Then, 2 mL of Rh6G2 stock solution (75 μM) were added to the reaction mixture at room temperature for 2 h to acquire GNPs-GSH-Rh6G2. GSH-Rh6G2 were prepared by a similar process, in which 150 μL of GSH stock solution (1 mM) was added into 2 mL Rh6G2 stock solution (75 μM), which was adjusted to pH 7 with NaOH (1 M). Nitrogen was used to protect the reaction for 2 h to acquire GSH-Rh6G2.

### Fluorescence measurements

The fluorescence emission intensity was measured at 560 nm with an excitation wavelength of 516 nm and the excitation and emission slits set were at 2.5 nm. 2.45 mL of GNPs-GSH-Rh6G2 was diluted to 4.00 mL with HEPES/CH_3_OH buffer solution (1:1 (v/v), 50 mM, pH 7). The solution of GNPs-GSH-Rh6G2 with metal ions was prepared by adding 30 μL of stock solution of Hg^2+^, Ag^+^, K^+^, Na^+^, Ca^2+^, Co^2+^, Cu^2+^, Fe^2+^, Mg^2+^, Mn^2+^, Pb^2+^, Zn^2+^, Al^3+^, and Fe^3+^ (0.75 mM). Each solution of GNPs-GSH-Rh6G2/ion was prepared in spectral cuvettes that were carefully cleaned to avoid contamination. Fluorescence spectra were measured after mixing well to fully interact with the GNPs-GSH-RH6G2. All tests were carried out in triplicate.

### Cell culture and imaging of intracellular molecular release

HeLa cells were incubated in Dulbecco's modified Eagle medium (DMEM) (the density is about 2 × 10^4^ cells per well) at 37 °C in a 5% CO_2_ for 48 h. After adding 100 μL of GSH-Rh6G2 and GNPs-GSH-Rh6G2 for 1 h, the cells were washed with HEPES buffer three times. Then, 30 μL of Hg^2+^ solution was added. Residual ions were washed with HEPES buffer before imaging. Confocal laser scanning microscopy with 543 nm excitation was carried out.

### Cytotoxicity assays

Cytotoxicity assays were used to investigate the bio-safety of GSH-Rh6G2 and GNPs-GSH-Rh6G2. HeLa cells with 8 × 10^3^ cells per well were incubated in a 96-well plate overnight. 100 μL of GSH-Rh6G2 and GNPs-GSH-Rh6G2 at different concentrations were added in DMEM medium at 37 °C in 5% CO_2_ for 24 h. The medium was replaced by 110 μL of 100 μL of EMEM medium completed with 10% fetal bovine serum (FBS) and 10 μL cell counting kit-8 reagent (CCK-8) and incubated for 2 h. Then, the samples were washed with HEPES buffer. A microplate reader was used to measure the absorbance at 450 nm. Each group had six parallel wells, and the experiment was repeated three times. The cells were calculated according to this equation: cell viability (%) = [*A*_450_ (sample) − *A*_450_ (blank)]/[*A*_450_ (control) − *A*_450_ (blank)] × 100%.

## Supporting Information

File 1 Additional experimental data.
