# Supplementary material for: Detection and imaging of Hg(II) in vivo using glutathione-functionalized gold nanoparticles
Source: Beilstein J Nanotechnol. 2022 Jun 23;13:549–59. doi: 10.3762/bjnano.13.46 (PMC9235832; doi:10.3762/bjnano.13.46)
Supplement: File 1 — Additional experimental data. [file Beilstein_J_Nanotechnol-13-549-s001.pdf]

BEILSTEIN JOURNAL OF NANOTECHNOLOGY

Supporting Information

for

Detection and imaging of Hg(II) in vivo using glutathione-functionalized gold nanoparticles

Gufeng Li, Shaoqing Li, Rui Wang, Min Yang, Lizhu Zhang, Yanli Zhang, Wenrong Yang and Hongbin Wang

Beilstein J. Nanotechnol. **2022,** *13,* 549–559. doi:10.3762/bjnano.13.46

Additional experimental data

License and Terms: This is a supporting information file under the terms of the Creative Commons Attribution License (https://creativecommons.org/licenses/by/4.0). Please note that the reuse, redistribution and reproduction in particular requires that the author(s) and source are credited and that individual graphics may be subject to special legal provisions.
The license is subject to the *Beilstein Journal of Nanotechnology* terms and conditions: (https://www.beilstein-journals.org/bjnano/terms)

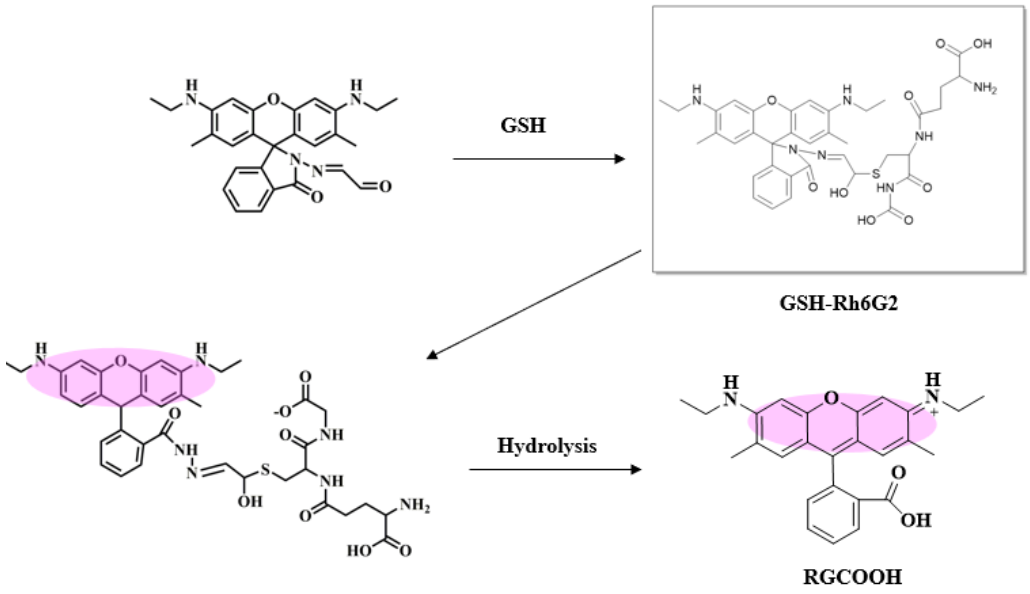

Figure S1: Speculative scheme of the reaction between Rh6G2 and GSH.

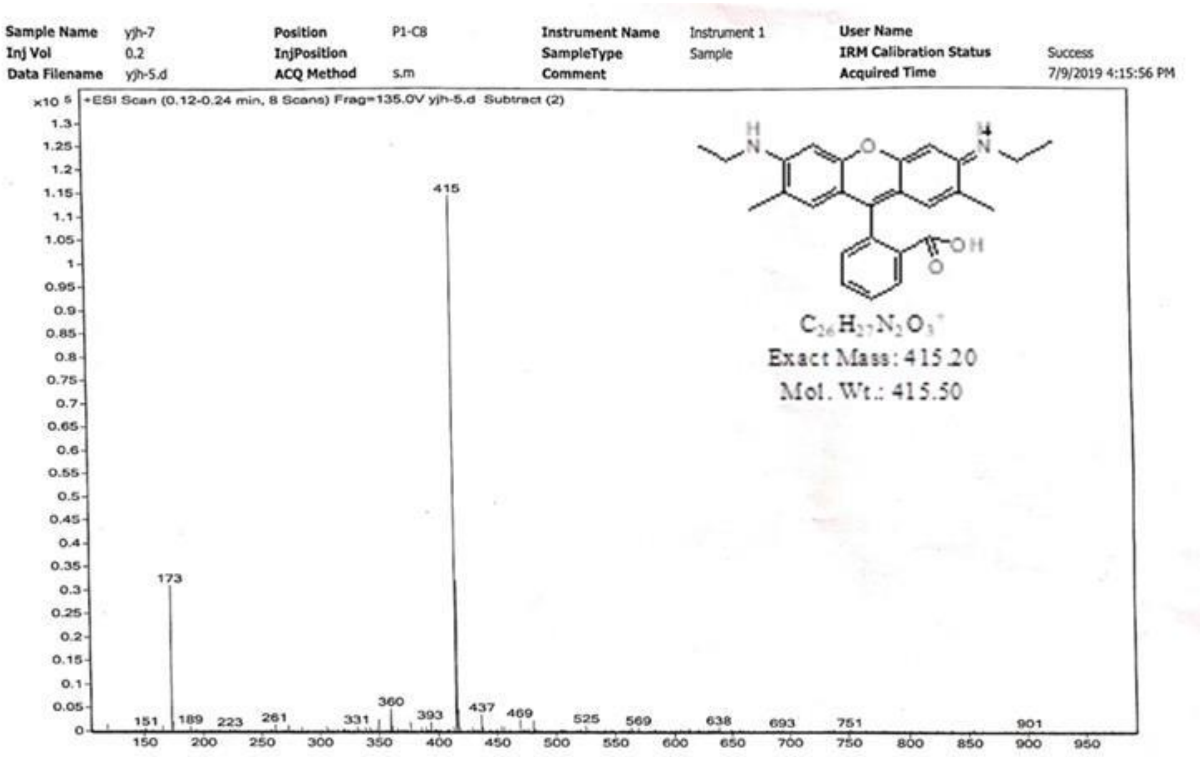

Figure S2: Mass spectra of GSH-Rh6G2. The fluorescent substance of GSH-Rh6G2 was separated, which proved that *m*/*z* is 415 as RGCOOH.

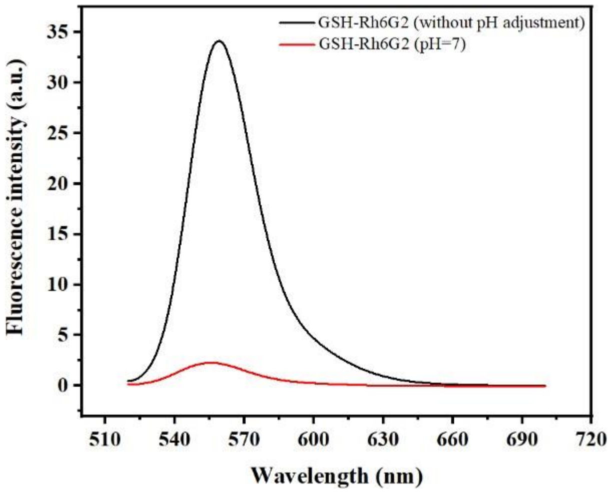

Figure S3: Fluorescence spectra of GSH-RH6G2 in weakly acidic environment (black curve) and in neutral environment (red curve).

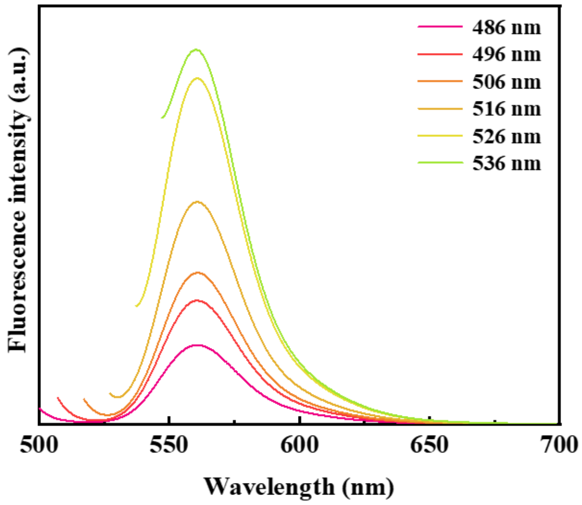

Figure S4: Fluorescence emission spectra of the GNPs-GSH-Rh6G2 with different excitation wavelengths.

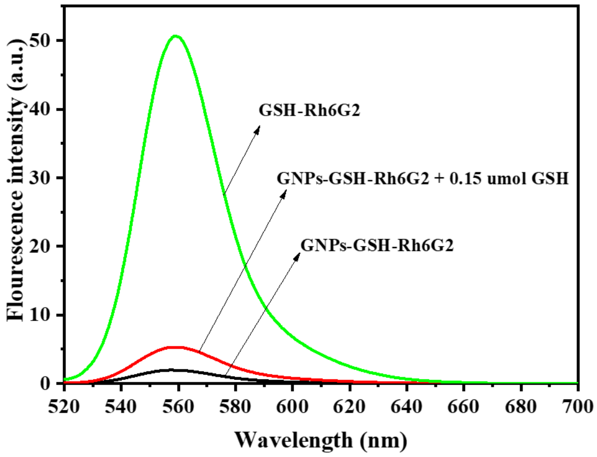

Figure S5: Fluorescence spectra of molecular saturation test of GNPs-GSH-RH6G2
